# Role of Human Body Composition Analysis and Malnutrition Risk Questionnaire in the Assessment of Nutritional Status of Patients With Initially Diagnosed Crohn's Disease

**DOI:** 10.3389/fmed.2020.00106

**Published:** 2020-04-09

**Authors:** Mrigul Kurban, Na Zeng, Meng Wang, Hong Liu, Jin-Ru Wu, Guo Feng, Min Liu, Qin Guo

**Affiliations:** ^1^Nutritional Department, Third Xiangya Hospital of Central South University, Changsha, China; ^2^Birth Cohort Study Department, Shunde Women and Children's Hospital of Guangdong Medical University, Foshan, China; ^3^Department of Gastroenterology, Third Xiangya Hospital of Central South University, Changsha, China

**Keywords:** Crohn's disease, nutrition assessment, body composition analysis, malnutrition, reduced muscle mass

## Abstract

**Objective:** This study was carried out to investigate the role and necessity of human body composition analysis in assessing the nutritional status of initially diagnosed Crohn's disease (CD) patients.

**Methods:** A total of 47 initially diagnosed CD patients were recruited. The skeletal muscle mass index (SMI), fat-free mass index (FFMI), body fat mass, body fat percent, visceral fat area (VFA), and body cell mass were determined with the Biospace Inbody S10 composition analyzer.

**Results:** In 47 patients with initially diagnosed CD, SMI could determine the muscular mass reduction that could not be determined by the body mass index (BMI) (35.3%), albumin (ALB) (65.6%), nutrition risk screening (NRS)2002 (25.0%), and Patient-Generated Subjective Global Assessment (PG-SGA) (55.6%). FFMI could determine the malnutrition that could not be determined by the BMI (58.8%), albumin (90.6%), NRS2002 (50.0%), and PG-SGA (55.6%). VFA in the fistulizing CD patients was significantly higher than in the stricturing and non-fistulizing, non-stricturing patients (*P* < 0.05). SMI and BMI had the same performance (*P* = 1.000) and general consistence (Kappa = 0.487, *P* = 0.001) in the assessment of malnutrition; SMI and ALB had different performance (*P* < 0.001) and inconsistence was noted (Kappa = 0.069, *P* = 0.489) in the assessment of malnutrition; the results of the nutrition assessment were different between SMI and NRS2002 (*P* = 0.002), and inconsistence was observed (Kappa = 0.190, *P* = 0.071). SMI and PG-SGA had the same performance in the assessment of nutrition (*P* = 0.143), but there was inconsistence (Kappa = 0.099, *P* = 0.464). FFMI and BMI had general consistence in the assessment of malnutrition (Kappa = 0.472, *P* < 0.001), but the positive rate determined by FFMI (85.1%) was markedly higher than that by BMI (63.8%) (*P* = 0.002). FFMI and ALB had different performance in the assessment of malnutrition (*P* < 0.001) and there was inconsistence (Kappa = −0.008, *P* = 0.877). FFMI and NRS2002 had the same performance in the assessment of malnutrition (*P* = 0.453), but the consistence was poor (Kappa = 0.286, *P* = 0.039). The results determined by SMI and PG-SGA were consistent (*P* = 0.727), but the consistence was poor (Kappa = 0.399, *P* = 0.006).

**Conclusion:** Human body composition analysis can identify the patients with muscular mass reduction that cannot be identified by commonly used nutrition assessment scales/parameters. Thus, it is helpful for the assessment of disease severity and also important for the nutrition assessment in CD patients.

## Introduction

Crohn's disease (CD) is a chronic granulomatous inflammatory disease of the whole intestine. The clinical manifestations of CD mainly include abdominal pain, diarrhea, fistula, perianal lesions, weight loss, and various degrees of systemic symptoms. CD is often affected by drugs, immunity, and food intake, and CD patients often have concomitant weight loss, anemia, and hypoproteinemia. The incidence of malnutrition is as high as 85% in CD patients (1). Currently, the nutrition status in CD patients is assessed mainly with nutrition assessment scales or parameters such as body mass index (BMI), albumin (ALB), nutrition risk screening 2002 (NRS2002), and Patient-Generated Subjective Global Assessment (PG-SGA).

The European Society for Parenteral and Enteral Nutrition (ESPEN) defines malnutrition as a state in which nutrient deficiency or inadequate intake leads to changes in the body composition [reduction of fat-free mass index (FFMI) or cell number], causing physical and mental dysfunction and affecting the clinical outcome of diseases (2). Sarcopenia can be diagnosed when the changes in body composition and muscular mass decrease cause the muscular strength decline. Studies have found that patients with chronic diseases (such as chronic obstructive pulmonary disease, heart disease, digestive tract cancer, hepatic cirrhosis, diabetes, or chronic renal insufficiency) usually have sarcopenia which will affect the outcome of chronic diseases (3–7). Recently, the parameters related to the human muscular mass and muscular strength are integrated into many diagnostic criteria for malnutrition (8).

In CD patients, the hormones and proinflammatory cytokines act together on some cellular pathways to decrease protein synthesis and increase protein decomposition, resulting in muscular mass reduction (9, 10). In addition, the protein intake reduces and/or the protein loss increases due to chronic or acute diarrhea in CD patients. Muscular mass reduction is one of the diagnostic criteria for sarcopenia. Studies have shown that sarcopenia is associated with poor clinical outcomes in CD patients. Sarcopenia may serve as an independent predictor of the surgical outcomes of patients with inflammatory bowel disease and is closely related to the increased incidence of postoperative complications. The CD patients with sarcopenia often have compromised muscular strength and quality of life (11, 12). The general nutrition screening tools cannot identify the muscle attenuation, and the harm of muscle mass reduction in the CD patients has not received extensive attention in China. The European Society for Parenteral and Enteral Nutrition defines malnutrition as a state in which nutrient deficiency or inadequate intake leads to changes in the body composition (reduction of FFMI or cell number), causing the physical and the mental dysfunction and affecting the clinical outcome of diseases (13). According to the Global Leadership Initiative on Malnutrition (GLIM) 2019, CD patients may be diagnosed with malnutrition when the skeletal muscle mass index (SMI) reduces (≤7 kg/m^2^ for men and ≤5.7 kg/m^2^ for women) (14). According to the Asian Expert Consensus on the Diagnosis and Treatment of Sarcopenia, the diagnosis of sarcopenia should be based on the reduction of muscular mass and compromised muscular function. Of note is the fact that SMI reduction is an important prerequisite (≤7 kg/m^2^ for men or ≤5.7 kg/m^2^ for women) in the diagnosis of sarcopenia. It can be seen that muscle mass-related SMI and FFMI have received increasing attention in body composition analysis. In China, the expert consensus on the inflammatory bowel disease recommends the use of NRS-2002 for the screening of nutritional risk and PG-SGA for the assessment of nutritional status. However, little is known about the role of NRS-2002 and PG-SGA in the assessment of muscular mass in CD patients. This study was undertaken to investigate the consistence of body composition parameters, which was determined by using the bioelectrical impedance method with the commonly used parameters for nutritional assessment (BMI, ALB, NRS2002, and PG-SGA), and to explore the role of body composition analysis in nutritional screening in CD patients.

## Materials and Methods

### Subjects

A total of 47 patients who were initially diagnosed with CD were recruited into the present study. There were 36 males and 11 females with the mean age of 31.04 ± 8.84 years. The inclusion criteria were as follows: (1) the patients were 18–90 years of age, (2) CD was diagnosed according to the diagnostic criteria for CD, (3) the patients were conscious and had no serious edema and ascites, (4) the height and the body weight could be accurately measured, and (5) the hospital stay was longer than 24 h and surgery was not performed during the hospitalization. This study has been approved by the Institutional Review Boards of the Third Xiangya Hospital, Central South University (2019-S540). The age on diagnosis, disease location, and disease behaviors were described according to the Montreal classification (15). According to the age on diagnosis, the patients were divided into A1 group (not older than 16 years), A2 group (17–40 years old), and A3 group (>40 years old). According to the location of the disease, the patients were divided into L1 group (ileal), L2 group (colonic), L3 group (ileocolonic), and L4 group (upper gastrointestinal tract). According to the disease behaviors, the patients were divided into B1 group (non-penetrating, non-stricturing), B2 group (stricturing), and B3 group (penetrating).

### Methods

The human body composition was estimated using a multifrequency bioimpedance analysis (BIA) with the InBody S10 analyzer (Biospace Co., Ltd., Seoul, Korea). InBody uses an eight-point tetrapolar electrode system method which assesses the impedance to six specific frequencies (1, 5, 50, 250, 500, and 1,000 kHz) and the impedance at three specific frequencies (5, 50, and 250 kHz) of a small alternate electrical current applied on the body.

The BIA measurements were performed by trained staff according to the standardized procedures. The measurements were conducted on the dominant side of the body (right side in most patients). The patients had an overnight fast, emptied the bladder by urinating, took off the clothes, and took a standing posture during the measurement, during which the ambient temperature remained at 25°C.

Skeletal muscle mass index = appendicular skeletal muscle mass/(height × height), fat-free mass index = fat free mass/(height × height), body fat mass (BFM) = body weight—fat free mass, body fat percent (BFP) = BFM/body weight × 100%, and body cell mass (BCM) = intracellular water + protein. Visceral fat area (VFA) was defined as a cross-sectional area of visceral fat in the abdomen at the umbilical level (L4–L5).

SMI of ≤7 kg/m^2^ for men or ≤5.7 kg/m^2^ for women was defined as low SMI (+). FFMI of <17 kg/m^2^ for men or <15 kg/m^2^ for women was defined as low FFMI (+). The height and the body weight were measured at shoes-free and fasting statuses, respectively, and the BMI was calculated. BMI of <18.5 kg/m^2^ was indicative of malnutrition (BMI+). Fasting blood was collected for the measurement of ALB. ALB of <30 g/L was defined as low ALB (+); NRS2002 was used for the screening of risk for nutrition, and a NRS2002 score ≥3 was suggestive of risk for nutrition (+) (16). Nutritional assessment was done with PG-SGA, and a PG-SGA score ≥4 was defined as malnutrition. The participants fasted between 8 am and 10 am, and venous blood was collected for the measurement of ALB.

### Statistical Analysis

Statistical analysis was performed with SPSS version 17.0 (Statistical Product and Service Solutions, CA, USA). FFMI, BCM, BMI, ALB, PG-SGA, SMI, BFM, BFP, VFA, and NRS2002 were subjected to test for normal distribution. The variables with normal distribution (FFMI, BCM, BMI, ALB, and PG-SGA) are expressed as mean ± standard deviation (SD); the variables with abnormal distribution (SMI, BFM, BFP, VFA, and NRS2002) are expressed as median + quartile. The FFMI and BCM were compared between patients with *t*-test; the BFM, BFP, VFA, and BCM were compared with Wilcoxon rank sum test. McNemer test and consistency analysis were employed to analyze the consistency of SMI and FFMI with NRS2002, PG-SGA, BMI, and ALB in the assessment of nutritional status. The relationship between SMI and BMI was determined with Spearman rank correlation analysis, and the relationship between FFMI and BMI was determined with Pearson correlation analysis. A value of *P* < 0.05 was considered as statistically significant.

## Results

### General Characteristics

A total of 47 patients with initially diagnosed CD were recruited into the present study. The mean SMI was 6.44 ± 1.05 kg/m^2^, and 66.0% of the patients had low SMI (≤7.0 kg/m^3^ for men and ≤5.7 kg/m^3^ for women). The mean FFMI was 15.07 ± 1.97 kg/m^2^, and low FFMI was noted in 85.1% of the patients (<17 kg/m^2^ for men and <15 kg/m^2^ for women). The mean fat-free body weight was 42.42 ± 7.53 kg, the mean BFM was 6.13 ± 3.58 kg, the mean VFA was 27.14 ± 18.77 cm^2^, and the mean BCM was 27.65 ± 5.09 kg. The mean BMI was 17.36 ± 2.56 kg/m^2^, and 63.8% of the patients had a BMI lower than 18.5 kg/m^2^. The mean ALB was 34.94 ± 6.81 g/L and 19.1% of the patients had ALB lower than 30 g/L. An assessment with NRS2002 showed a score of 3 in eight patients (17.0%), a score of 4 in 23 patients (48.9%), and a score of 5 in 12 patients (25.5%), and 91.5% of the patients had a risk for malnutrition; an assessment with PG-SGA showed a score of 2–3 in six patients (12.8%), a score of 4–8 in 27 patients (57.4%), and a score of ≥9 in 11 patients (23.4%); 12.8% of the patients had suspected malnutrition, and 80.9% of the patients had malnutrition (Table 1). In the present study, the types of CD included A2 (91.5%), L3 (51.1%), and B1 (42.6%) (Table 2).

**Table 1:** Nutritional status and human body composition of Crohn’s disease patients.

**Parameters**	**x+*s***	**Parameters**	**x+*s***
Height (cm)	167.32 ± 6.93	SMI (kg/m^2^)	6.59 ± 0.71
Weight (kg)	48.78 ± 8.95	FFMI (kg/m^2^)	15.07 ± 1.97
BMI (kg/m^2^)	17.36 ± 2.56	BFM (kg)	5.50 ± 3.20
ALB	34.94 ± 6.81	BFP (%)	11.1 ± 4.70
NRS2002 score		VFA (cm^2^)	26.1 ± 9.20
3	8 (17.0)	BCM (kg)	27.65 ± 5.09
4	23 (48.9)		
5	12 (25.5)		
PG-SGA score			
2–3	6 (12.8%)		
4–8	27 (57.4%)		
≥9	11 (23.4%)		

*SMI, skeletal muscle mass index; FFMI, fat-free mass index; BFM, body fat mass; BFP, body fat percent; VFA, visceral fat area; BCM, body cell mass; ALB, albumin*.

**Table 2:** Characteristics of Crohn’s disease patients.

**Age**	***n* (%)**	**Location**	***n* (%)**	**Type**	***n* (%)**
A1	—	L1	15 (31.9)	B1	20 (42.6)
A2	45 (91.5)	L2	8 (17.0)	B2	14 (29.8)
A3	2 (8.5)	L3	24 (51.1)	B3	13 (27.7)

*A1, <16 years; A2, 16–40 years; A3, >40 years; L1, terminal ileal; L2, colonic; L3, ileocolonic; B1, inflammatory; B2, stricturing; B3, penetrating*.

### Human Body Composition in Patients With Different Characteristics

The SMI, FFMI, and BCM in males were significantly higher than in females, but the BFP in females was markedly higher than in males (*P* < 0.05). The SMI, FFMI, BFM, BFP, and BCM were similar among patients with CD at different locations and of different types. The VFA in fistulizing CD patients was significantly larger than in stricturing CD and non-fistulizing, non-stricturing patients (*P* < 0.05) (Table 3).

**Table 3:** Human body composition in patients with different characteristics.

**Variables**	**SMI**	**FFMI**	**BFM**	**BFP**	**VFA**	**BCM**
Gender						
M	6.88 ± 0.96	15.72 ± 1.70	5.50 ± 5.03	10.80 ± 7.98	23.70 ± 23.55	29.76 ± 3.72
F	4.93 ± 0.56^*^	12.93 ± 1.06^*^	5.60 ± 5.20	15.5 ± 11.10^*^	26.90 ± 9.50	20.75 ± 1.49^*^
Location						
L1	7.26 ± 1.04	15.90 ± 1.80	5.80 ± 5.10	11.40 ± 5.70	26.4 ± 22.90	29.61 ± 4.50
L2	6.50 ± 1.45	14.65 ± 1.42	5.25 ± 2.08	11.35 ± 5.73	23.35 ± 20.08	25.98 ± 3.59
L3	6.40 ± 2.15	14.68 ± 2.12	5.00 ± 6.00	10.95 ± 9.23	26.55 ± 22.10	26.98 ± 5.61
Type of disease						
B1	6.46 ± 1.72	14.88 ± 1.80	4.90 ± 3.95	10.35 ± 7.30	19.10 ± 18.43	27.39 ± 5.00
B2	6.55 ± 2.12	14.96 ± 1.92	5.20 ± 4.55	11.50 ± 9.85	24.20 ± 23.28	26.98 ± 5.04
B3	6.85 ± 1.22	15.47 ± 2.34	6.80 ± 4.70	11.80 ± 5.65	35.30 ± 15.55^a^^,^ ^b^	28.78 ± 5.47

*A1, <16 years; A2, 16–40 years; A3, >40 years; L1, terminal ileal; L2, colonic; L3, ileocolonic; B1, inflammatory; B2, stricturing; B3, penetrating*.

* *P < 0.05 vs. males*.

a *P < 0.05: B1 vs. B3*.

b *P < 0.05: B2 vs. B3*.

### Consistence Between the Results of Human Body Composition and the Results of Nutritional Assessment With Scales/Nutritional Parameters

There was consistence between SMI and BMI in the assessment of malnutrition (*P* = 1.000), and the consistence was general (Kappa = 0.487, *P* = 0.001). Inconsistence was noted between SMI and ALB in the assessment of malnutrition (*P* < 0.001), and there was no consistence (Kappa = 0.069, *P* = 0.489). Inconsistence was noted between SMI and NRS2002 in the assessment of malnutrition (*P* = 0.002), and there was no consistence (Kappa = 0.190, *P* = 0.071). SMI and PG-SGA had consistence in the assessment of malnutrition (*P* = 0.143), but there was no consistence (Kappa = 0.099, *P* = 0.464) (Table 4).

**Table 4:** Consistence of the skeletal muscle mass index and the fat-free mass index with the nutritional scales/parameters in the nutritional assessment.

**BIA**	**BMI**		**ALB (41, missing** **=** **6)**		**NRS2002**		**PG-SGA**		**Total**
	**+**	**–**	**+**	**–**	**+**	**–**	**+**	**–**	
SMI(+)	25 (83.3)	6 (35.3)	7 (77.8)	21 (65.6)	30 (69.8)	1 (25.0)	26 (68.4)	5 (55.6)	31 (66.0)
SMI(-)	5 (16.7)	11 (64.7)	2 (22.2)	11 (34.4)	13 (30.2)	3 (75.0)	12 (31.6)	4 (44.4)	16 (34.0)
FFMI(+)	30 (100.0)	10 (58.8)	8 (88.9)	29 (90.6)	38 (88.4)	2 (50.0)	35 (92.1)	5 (55.6)	40 (85.1)
FFMI(-)	0 (0.0)	7 (41.2)	1 (11.1)	3 (9.4)	5 (11.6)	2 (50.0)	3 (7.9)	4 (44.4)	7 (14.9)
Total	30 (63.8)	17 (36.2)	9 (22.0)	32 (78.0)	43 (91.5)	4 (8.5)	38 (80.9)	9 (19.1)	47 (100.0)

*ALB, albumin; BIA, bioimpedance analysis; BMI, body mass index; FFMI, fat-free mass index; NRS2002, nutrition risk screening 2002; PG-SGA, Patient-Generated Subjective Global Assessment; SMI, muscle mass index*.

The consistence between FFMI and BMI in the assessment of malnutrition was general (Kappa = 0.472, *P* < 0.001), but the positive rate determined by FFMI (85.1%) was significantly higher than that by BMI (63.8%) (*P* = 0.002). FFMI and ALB had inconsistence in the assessment of malnutrition (*P* < 0.001), and there was no consistence (Kappa = −0.008, *P* = 0.877). FFMI and NRS2002 had consistence in the assessment of nutritional status (*P* = 0.453), but the consistence was poor (Kappa = 0.286, *P* = 0.039). FFMI and PG-SGA had consistence in the assessment of nutritional status (*P* = 0.727), but the consistence was poor (Kappa = 0.399, *P* = 0.006) (Table 4).

### Correlations of SMI and FFMI With BMI

Consistence was noted among SMI, FFMI, and BMI in the assessment of nutritional status. A further correlation analysis showed a positive relationship between SMI and BMI (*r*_*s*_ = 0.681, *P* < 0.001). Moreover, a positive relationship was noted between SMI and BMI in males (*r*_*s*_ = 0.786, *P* < 0.001), but there was no correlation in females (*r*_*s*_ = 0.518, *P* = 0.102). There was a positive relationship between FFMI and BMI (*r* = 0.824, *P* < 0.001). Moreover, this positive relationship was noted in both males (*r* = 0.905, *P* < 0.001) and females (*r* = 0.663, *P* = 0.026).

## Discussion

CD is a systemic disease caused by genetic and environmental factors. The persistent chronic inflammation and gastrointestinal symptoms such as diarrhea and loss of appetite are likely to cause malnutrition and changes in body composition. Nutritional support is an important treatment for CD. Early detection of malnutrition, early initiation of nutritional therapy, and regular assessment of the nutritional status of patients with risk for malnutrition can improve the outcomes (17). The nutritional screening scales/parameters currently widely used in China play important roles in the assessment of malnutrition. Although the GLIM guideline (14) for the first time recommends the assessment of muscular mass reduction, it has not been confirmed in prospective clinical trials and its relationship with clinical outcomes has never been validated. According to the GLIM criteria, malnutrition can be diagnosed when at least one phenotypic criterion and one etiological criterion are present. The phenotypic criteria for malnutrition include weight loss, BMI reduction, and reduction of muscular mass; the etiological criteria for malnutrition include reduced food intake or absorption and inflammatory status. CD patients have persistent inflammatory status, which is one of the etiological criteria for malnutrition in the GLIM guideline. Thus, malnutrition can be diagnosed once the weight loss, the BMI reduction, or the reduction of muscular mass reaches the corresponding threshold. In CD patients, increasing attention has been paid to weight loss and BMI reduction, but the reduction of muscular mass has not been widely accepted as an indicator of malnutrition, and few studies have been conducted to investigate this issue (18). Sarcopenia is a disease status reflecting the reduction of muscular mass and the compromise of muscular function. Increasing studies have shown that sarcopenia is closely related to the poor outcome of CD. The reduction in muscular mass is one of the important diagnostic criteria for sarcopenia. Thus, in recent years, some clinicians have emphasized the role of muscular mass reduction in the assessment of malnutrition. SMI and FFMI may reflect the muscular mass and can be obtained by detecting body composition.

Currently, little is known about the role of body composition analysis in the nutritional assessment of patients with CD. Computed tomography (CT), magnetic resonance imaging, and dual-energy X-ray absorptiometry (DEXA) are the gold standards in the assessment of muscular mass. Furstenberg and Davenport. found that BIA had high consistence with DEXA in the assessment of muscular mass. Moreover, BIA is a safe and simple examination that can be done rapidly, and thus it has been widely used in clinical practice. In the present study, BIA was employed to determine the SMI and FFMI of patients initially diagnosed with CD (19).

Our study showed that patients with newly diagnosed CD had poor nutritional status. On screening with NRS2002, 91.5% of the patients had the risk for malnutrition; on screening with PG-SGA, 80.9% of the patients had malnutrition. The average BMI was 17.36 ± 2.56 kg/m^2^, and 63.8% of the patients had a BMI <18.5. Low SMI was observed in 66.0% of the patients, and 85.1% of the patients had low FFMI. Both human body composition analysis and assessment with nutritional scales showed that more than 50% of CD patients had malnutrition. Hypoproteinemia is associated with multiple disease outcomes such as post-operative wound healing and complications in patients with CD (20), and 19.1% of the patients with newly diagnosed CD had hypoproteinemia. The body composition analysis showed that there were no significant differences in SMI, FFMI, BFM, BFP, and BCM among patients with CD at different sites. This suggests that, although the absorption of nutrients mainly occurs in the small intestine, lesions in the colon or ileocecal area may also cause malnutrition, which may be related to the exhaustive status and systemic inflammation.

**Figure 1:**
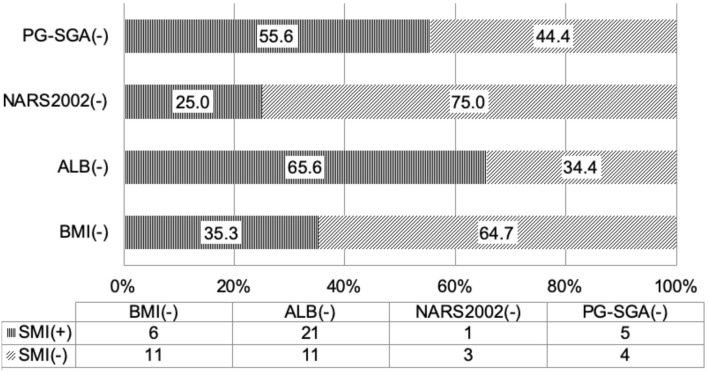
Consistence of the skeletal muscle mass index with the nutritional scales/parameters in the nutritional assessment of Crohn's disease patients.

According to the diagnostic criteria of the GLIM guideline, when the nutritional status was assessed based on SMI, 1/4 of the patients who had no risk for malnutrition on NRS2002 screening had low SMI, suggesting malnutrition; more than 50% of patients who had normal nutritional status on PG-SGA assessment had low SMI (Table 3 and Figure 1), suggesting malnutrition. Thus, some patients who have no risk for malnutrition or have normal nutritional status on screening with traditional scales may be diagnosed with malnutrition on the basis of body composition analysis. The use of nutritional status scales (such as NRS2002 and PG-SGA) in the screening of risk for malnutrition or assessment of malnutrition may cause a missed diagnosis of malnutrition in a majority of patients. The reduced muscular mass is an important phenotype in the phenotypic criteria for malnutrition according to the GLIM. CD patients should receive nutritional support once low SMI is present. FFMI is recommended for the assessment of malnutrition according to the ESPEN. Malnutrition may be diagnosed if patients with weight loss had low FFMI. There is evidence showing that the FFMI tends to reduce with the prolongation of duration of disease in the CD patients (9). Nutritional support should be administered in patients who have inflammatory status and reduced muscular mass, and nutritional support is beneficial for CD patients (21–23). In patients who had no risk for malnutrition on NRS2002 screening, about 50% had low FFMI (<17 kg/m^2^ for men and <15 kg/m^2^ for women); in patients who had normal nutritional status on PG-SGA assessment, more than 50% had low scores (Table 3 and Figure 1). Our results showed that 35.3% of the patients with normal BMI had low SMI and 58.8% had low FFMI. Thus, we speculate that, in the absence of body composition analysis, some patients initially diagnosed with CD should receive nutritional intervention. However, these patients do not receive timely nutritional support due to the limitations of the nutritional assessment scales. In clinical practice, the clinical nutritional screening, the nutritional assessment, and the BMI should be treated flexibly in CD patients. The normal nutritional status on screening with nutritional assessment scales, nutritional assessment, and BMI-based assessment do not mean that the patients do not need nutritional intervention.

According to the Asian Expert Consensus on the Diagnosis and Treatment of Sarcopenia, the diagnosis of sarcopenia should be based on the reduction of muscular mass and compromise of muscular function, and low SMI is an important prerequisite in the diagnosis of sarcopenia, especially severe sarcopenia (24). In the present study, the patients with low SMI accounted for as high as 67% (31/47), indicating that some CD patients might have sarcopenia. Thus, the CD patients may benefit from the detection of muscular mass and/or muscular function.

In CD patients, the white fat tissues of the mesentery close to the lesioned intestine are also known as “crawling fat” and associated with the pathogenesis of CD (25–27). Erhayiem et al. found that the patients with strictures or fistulas had a higher ratio of visceral fat to subcutaneous fat than those with only intestinal inflammation (28). Li et al. investigated the VFA of the CD patients by CT and found that a high VFA was associated with clinical and endoscopic early recurrence after surgery for CD (29), and VFA was an independent risk factor for post-operative recurrence (30). Our study showed that the VFA in fistulizing CD patients was significantly larger than in non-fistulizing, non-stricturing CD patients and stricturing CD patients. Thus, we speculated that VFA may be used to predict the disease severity, but further investigation is needed.

**Figure 2:**
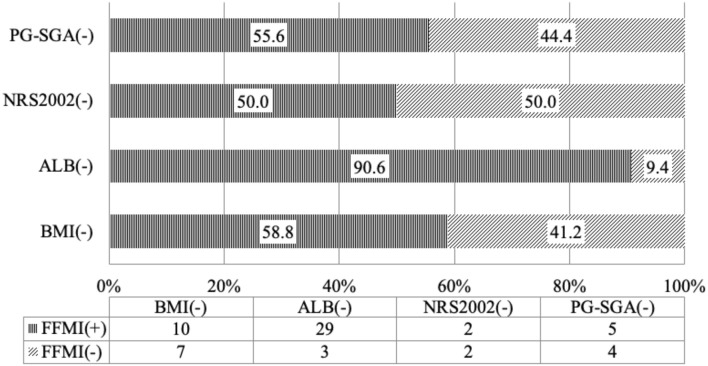
Consistence of the fat-free mass index with the nutritional scales/parameters in the nutritional assessment of Crohn's disease patients.

Our findings indicated that the results after assessment of malnutrition with traditional tools were different from those with the method based on body composition. Thus, further consistency analysis was conducted between the two methods (Figure 2). The results showed that NRS2002 and SMI had inconsistence in the assessment; although PGS-SGA and SMI had consistence in the assessment, there was no consistence; and although FFMI had consistence with NRS2002 and PG-SGA in the malnutrition assessment, the consistence was poor. These findings suggested that, although NRS2002 and PGS-GSA are important, commonly used tools for the nutritional assessment by dietitians, they may cause a missed diagnosis of malnutrition in some patients who need nutritional support, and thus they cannot replace the SMI or the FFMI in the evaluation of sarcopenia. ESPEN defines malnutrition as a combination of BMI (<18.5 kg/m^2^) reduction and weight loss or a combination of BMI reduction and FFMI reduction. Our results showed that FFMI was consistent with SMI in the nutritional assessment, but the consistence was poor. In the assessment with FFMI, the effect of fat on the muscular mass is excluded, but FFMI is still affected by other body components such as bones. Therefore, although FFMI reflects the muscular mass of patients to a certain extent, SMI may reflect the muscular mass more accurately and is more useful in the evaluation of sarcopenia.

Our study showed that SMI and BMI were consistent in the assessment, but the consistence was general, and BMI had limitations in the assessment of muscular mass. BMI is calculated based on body weight (in kg) and body height. The body weight has involvement of bones, fat, muscles, and other components. In the population with normal BMI, the muscular mass reduction may be still present due to the proportional imbalance of the body components. Bryant et al. (31) found that the obesity in obese patients with CD increased with the muscular mass reduction. In clinically treated patients with CD, an elevated BMI does not necessarily mean an improvement of disease condition, and body composition analysis should be done to avoid BMI increase due to water content increase (edema), body fat elevation, and muscular mass reduction. Clinicians should pay attention to the patients with normal BMI, aiming to avoid the delayed diagnosis and treatment. A further correlation analysis showed the positive relationship between SMI and BMI in males, but not in females. We speculate that the BFP is lower in males than in females and thus the muscular mass in males is closer to the BMI. ALB and SMI had inconsistence in the assessment, but there was no consistence; 65.5% of ALB-negative patients still had muscular mass reduction. Thus, the importance of ALB in the evaluation of sarcopenia is questioned in CD patients.

### Limitations of the Study

The limitations of our study are the relatively heterogeneous population and the small sample size in the subgroup analysis. Although our study was conducted in a referral center for IBD, our outpatient population is heterogeneous and the patients are from different parts of the country. In the future, more prospective studies with a large sample size are needed to confirm our findings.

## Conclusions

Taken together, body composition analysis can provide objective and sensitive indicators for the malnutrition assessment in initially diagnosed CD patients. Body composition analysis plays an important role in the nutritional assessment of patients with initially diagnosed CD, which cannot be replaced with NRS2002, PG-SGA, BMI, ALB, and other parameters/scales used for nutritional assessment. The screening and the assessment of malnutrition in CD patients should be individualized based on multiple factors such as patient's age, disease status, nutritional screening scale, and body composition analysis, which requires the cooperation between dietitians and gastroenterologists.

## Data Availability Statement

The raw data supporting the conclusions of this article will be made available by the authors, without undue reservation, to any qualified researcher.

## Ethics Statement

The studies involving human participants were reviewed and approved by This study has been approved by the institutional review boards of Third Xiangya Hospital, Central South University (2019-S540). The patients/participants provided their written informed consent to participate in this study.

## Author Contributions

ML and QG designed this study. MK and NZ performed the research, analyzed the data, and wrote the paper. MK, NZ, MW, HL, J-RW, GF, ML, and QG collected all the data. All authors read and approved the final version of this manuscript.

### Conflict of Interest

The authors declare that the research was conducted in the absence of any commercial or financial relationships that could be construed as a potential conflict of interest.
